# Predicting the Outcome of Sick Neonates Using the Sick Neonate Score: A Prospective Observational Study

**DOI:** 10.7759/cureus.105833

**Published:** 2026-03-25

**Authors:** Mohd Ashraf, Asma Wani, Parvez Ahmed, Waseem Shafi Sheikh, Mohsin Rashid

**Affiliations:** 1 Pediatric Nephrology, Government Medical College, Srinagar, IND; 2 Paediatrics, Government Medical College, Srinagar, IND; 3 Pediatrics, Government Medical College, Srinagar, IND

**Keywords:** neonatal intensive care unit (nicu), neonatal mortality, neonatal transport, respiratory distress syndrome (rds), sick neonate score

## Abstract

Background: At the critical transition from intrauterine to extrauterine life, prompt and precise management by the healthcare team is of immense importance. Neonatal transport services play a vital role during this period. The aim of this study was to evaluate the association between the Sick Neonate Score (SNS) and mortality among extramural neonates admitted to the neonatal ICU (NICU).

Methods: This prospective observational study was conducted from November 2019 to October 2021. All extramural neonates admitted to the NICU were enrolled, excluding those with surgical conditions, congenital anomalies, or syndromic features. Demographic details of the neonate and mother, indications for referral, mode of transport, and the presence of accompanying trained personnel were documented. Each neonate was followed until discharge or death. The condition at arrival was assessed, and the SNS was used to predict outcomes.

Results: A total of 422 neonates met the inclusion criteria, with a mortality rate of 115 (27.3%). The most common reasons for referral were respiratory distress syndrome 119 (28.2%), hyperbilirubinemia 91 (21.6%), and sepsis 84 (19.9%). The SNS demonstrated high predictive value for mortality, with a sensitivity of 106/115 (91.3%), specificity of 254/307 (82.7%), and an area under the receiver operating characteristic (ROC) curve (AUC) of 0.913. A cut-off score of ≤7 was associated with a significantly poorer prognosis compared to scores >7.

Conclusion: The SNS is simple to comprehend, easy to use, and requires minimal technology or expertise.

## Introduction

It is estimated that more than two-thirds of annual neonatal deaths occur in underdeveloped countries with limited resources, with India alone contributing to one-fourth of the global burden [1,2]. The most common causes of neonatal mortality include infections, birth asphyxia, prematurity, intrauterine growth restriction, meconium aspiration syndrome, and hyaline membrane disease. Alarmingly, approximately 40% of neonatal deaths occur on the first day of life [3,4]. This high early neonatal mortality could be significantly reduced with timely and appropriate medical interventions, which are often available only in capital or metropolitan cities.

A substantial proportion of deliveries still occur at home in non-institutional and non-scientific settings, increasing the risk of perinatal insults and complications, and thus necessitating the transport of newborns to specialized centers. The condition of the neonate at birth, timely recognition of the need for referral, and the circumstances under which the neonate is transported are critical determinants of survival and long-term outcomes. Stabilization before transport and maintenance of a stable microenvironment during transfer are essential for optimizing neonatal outcomes. The effectiveness of neonatal transport is influenced by the mode of transport, availability of trained personnel, appropriate equipment, and necessary medications. Any inadequacies during transport can significantly increase morbidity and mortality in this vulnerable population [5].

Several scoring systems have been developed to assess the condition and predict the outcomes of critically ill neonates, including the Score for Neonatal Acute Physiology (SNAP), Score for Neonatal Acute Physiology-Perinatal Extension (SNAPPE II), Clinical Risk Index for Babies (CRIB), and Neonatal Therapeutic Intervention Scoring System (NTISS) [6-9]. However, these scores often require advanced equipment and trained personnel, and are not feasible in low-resource settings. The Sick Neonate Score (SNS), in contrast, is a simple and practical clinical scoring system that requires minimal technology and expertise, making it particularly suitable for such environments.

In the peripheral regions of the Kashmir Valley and adjoining areas of the Jammu province, the population is predominantly rural, and tertiary neonatal care centers are limited compared to the central city of Srinagar. Neonatal transport in these areas is often undertaken using private cars, buses, motorcycles, bicycles, or tricycles. Given these challenges, the present study was designed to evaluate the utility of the SNS in assessing the condition of neonates at arrival and its association with neonatal mortality in our resource-limited setting.

## Materials and methods

A prospective observational study was conducted from November 2019 to October 2021 after obtaining approval from the Institutional Ethics Committee (Government Medical College, Srinagar, Jammu and Kashmir; Approval No. IRBGMC/PEDIA 351, dated May 22, 2019). The study was carried out in the neonatal division of the Department of Pediatrics at the erstwhile GB Pant Hospital (now a 500-bedded Children’s Hospital), an associated tertiary care referral center of Government Medical College, Srinagar, catering to the pediatric population of the Kashmir Valley.

All extramural neonates (aged ≤28 days) admitted to the neonatal ICU (NICU) were enrolled in the study, excluding those with surgical conditions, congenital anomalies, or syndromic features.

Upon arrival, detailed documentation was performed by a senior resident in the NICU using a predesigned proforma, including demographic data of the neonates and their mothers, indications for referral, mode of transport, and whether trained personnel accompanied the neonate during transfer. The neonates were then followed until discharge or death.

Neonatal demographic parameters recorded included gestational age, mode of delivery, need for resuscitation at birth, APGAR score (Appearance (skin color), Pulse (heart rate), Grimace (reflex irritability), Activity (muscle tone), and Respiration), age at arrival, gender, and birth weight. Maternal details, such as history of premature rupture of membranes (PROM), cardiac disease, antenatal steroid use, gestational diabetes mellitus (GDM), pregnancy-induced hypertension (PIH), and seizure disorders, were also documented.

Additionally, transport-related details were collected, including indications for referral (Table 1), mode of transport, and use of medications or procedures during pretransport and intratransport phases. Upon arrival and before starting any treatment modality, clinical parameters such as heart rate, respiratory effort, capillary refill time, body temperature, mean arterial pressure, capillary blood glucose, and oxygen saturation were recorded.

**Table 1:** Various indications for referral to the tertiary care centre (n=422) Data presented as absolute numbers (n) and percentages (%). LBW: Low birth weight

Indications	n (%)
Asphyxia	42 (10.0)
Sepsis	84 (19.9)
Preterm or LBW	36 (8.5)
Hypoglycemia	10 (2.4)
Respiratory distress syndrome	119 (28.2)
Seizures	7 (1.7)
Neonatal hyperbilirubinemia	91 (21.6)
Cardiac	12 (2.8)
Others	21 (5.0)

Perinatal asphyxia was defined by hypoxia and hypercapnia occurring at or around the time of delivery, resulting from lack of oxygen or ischemia that is prolonged or severe enough to cause harmful effects on the fetal brain, heart, lungs, or kidneys [10,11]. Neonatal sepsis was defined as a life-threatening, dysregulated inflammatory response to bloodstream infection in infants less than 28 days old [12]. Neonatal hypoglycemia was defined as a neonatal blood glucose concentration of < 2.6 mmol/L [13]. Neonatal hypothermia was defined as a core body temperature <36.5°C in a newborn [14].

After calculating the SNS at the time of admission, statistical analysis was performed using Microsoft Excel for Windows (version 2009). Using SNS as the test variable, a receiver operating characteristic (ROC) curve was generated, with a cut-off score of ≤7 demonstrating 100% specificity. A binary logistic regression analysis was performed with in-hospital mortality as the dependent variable and SNS as the independent variable to assess its association with outcome. No additional covariates were included, given the exploratory nature of the analysis. Two-sided p-values were reported, and a p-value of <0.05 was considered statistically significant.

## Results

**Figure 1:**
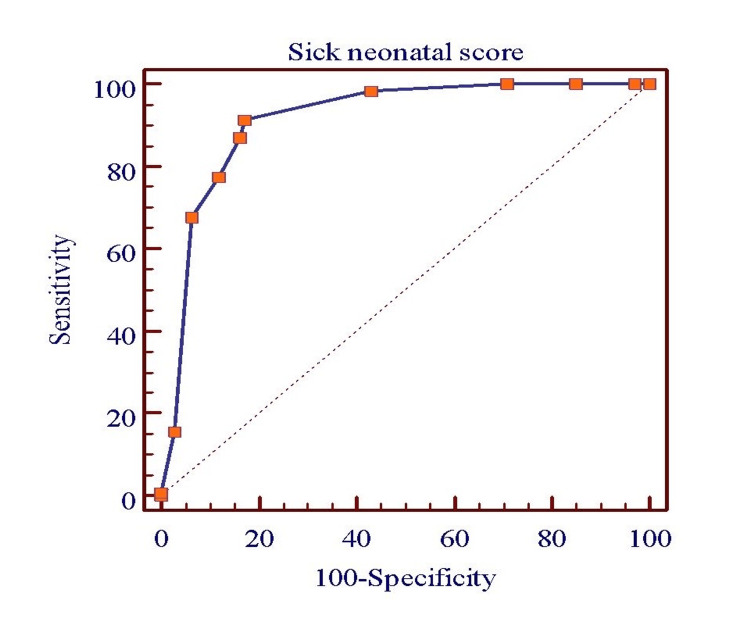
ROC curve analysis depicting diagnostic accuracy of SNS in predicting mortality ROC: Receiver operating characteristic; SNS: Sick Neonate Score

During the study period, 500 neonates were assessed for eligibility, of whom 422 (84.4%) met the inclusion criteria and were enrolled as the study population. The SNS was calculated for all enrolled patients (Table 2) [15]. Among the 115 neonates who expired, 97 (84.3%) had an SNS ≤7, while 18 (15.7%) had an SNS >7 (p < 0.001) (Table 3). Of the neonates who died within 24 hours of admission, more than 90% were hypothermic (Table 4). After scoring was completed using the SNS, a ROC curve demonstrated that a cut-off score of ≤7 was predictive of mortality (Figure 1).

**Table 2:** Outcome of study neonates (n=422)

Outcome	n (%)
Discharged	307 (72.7)
Death	115 (27.3)
Total	422 (100)

**Table 3:** SNS [15] SNS: Sick Neonate Score

Variable	Score		
	0	1	2
Respiratory effort	Apnea or grunting	Tachypnea (>60/min) with respiratory distress	Normal (40-60/min)
Heart rate	Bradycardia or asystole	Tachycardia (>160/min)	Normal (100-160/min)
Mean blood pressure (mmHg)	<30	30-39	>39
Axillary temperature (°C)	<36	36-36.5	36.5-37.5
Capillary refill time (seconds)	>5	3-5	<3
Random blood sugar (mg/dl)	<40	40-60	>60
Oxygen saturation (SpO_2_) on room air	<85	85-92%	>92%

**Table 4:** Association of measured variables at arrival and outcome Data are presented as numbers (n) and percentages (%). P-values were calculated using the chi-square (χ²) test. Statistical significance was defined as p < 0.05.

Variable	Range	Discharged n(%)	Death n(%)	Chi-square value	P-value	Significance
Respiratory rate (per minute)	<40	59 (19.2%)	72 (62.6%)			
	40–60	139 (45.3%)	2 (1.7%)	98.20	<0.001	Significant
	>60	109 (35.5%)	41 (35.7%)			
Heart rate (per minute)	<110	0 (0.0%)	12 (10.4%)			
	110–160	236 (76.9%)	20 (17.4%)	135.98	<0.001	Significant
	> 160	71 (23.1%)	83 (72.2%)			
Temperature (°C)	<36	16 (5.2%)	25 (21.7%)			
	36–36.5	241 (78.5%)	87 (75.7%)	36.07	<0.001	Significant
	36.5–37.5	50 (16.3%)	3 (2.6%)			
Capillary refill time (seconds)	> 5	12 (3.9%)	23 (20.0%)			
	3–5	257 (83.7%)	90 (78.3%)	36.41	<0.001	Significant
	<3	38 (12.4%)	2 (1.7%)			
Mean blood pressure (mmHg)	<30	26 (8.5%)	29 (25.2%)			
	30–39	233 (75.9%)	85 (73.9%)	46.41	<0.001	Significant
	> 39	48 (15.6%)	1 (0.9%)			
Random blood sugar (mg/dl)	<40	36 (11.7%)	61 (53.0%)			
	40–60	192 (62.5%)	45 (39.1%)	66.52	<0.001	Significant
	> 60	79 (25.7%)	9 (7.8%)			
Oxygen saturation (SpO_2_)	<85	15 (4.8%)	24 (20.8%)			
	85–92%	242 (78.8%)	88 (76.5%)	32.66	<0.001	Significant
	> 92%	50 (16.2%)	3 (2.6%)			

## Discussion

Neonatal transport is one of the important factors that determine the clinical outcome of extramural sick neonates, yet it is often neglected in developing countries like ours. The major indications for neonatal referral to our hospital were respiratory distress syndrome 119 (28.2%), neonatal jaundice 91 (21.6%), and sepsis 84 (19.9%). Other indications included birth asphyxia, hypoglycemia, and prematurity or low birth weight (LBW). These findings are consistent with earlier studies [16,17]. Data from the National Neonatal-Perinatal Database (NNPD), a multicenter neonatal morbidity and mortality surveillance initiative in India, also identified sepsis, birth asphyxia, and prematurity as the leading indications for referral to higher centers [18]. In contrast, in developed countries, extreme prematurity and congenital malformations are the predominant reasons for neonatal referrals [19].

In our study, the SNS incorporated additional parameters, such as capillary refill time, oxygen saturation (SpO₂), respiratory effort, and heart rate, making it simple to use, reproducible, and clinically relevant. A cut-off score of ≤7 was found to predict poor outcomes effectively. All components of the SNS showed a statistically significant correlation with clinical outcomes, where a lower score was associated with a higher risk of mortality, a finding that aligns with the study by Rathod et al. [15].

Furthermore, neonates who were transported by ambulance demonstrated better survival rates and a lower incidence of hypothermia and hypoglycemia compared to those transported by other means. This observation is consistent with the findings of Modanlou et al. [20]. Additionally, Spector et al. observed that neonatal mortality in low-resource settings can be reduced through structured checklists and pretransport protocols [21]. Another study done by Singh et al. observed that paucity of trained staff during transport, lack of prereferral stabilization, and travel time more than two hours were some important predictors of neonatal mortality [22].

We observed that poor respiratory effort or a low respiratory rate (bradypnea), along with low mean blood pressure, was associated with adverse outcomes (Table 4). This finding highlights the vital importance of maintaining TABC (Temperature, Airway, Breathing, and Circulation) during neonatal transport.

Respiratory distress affects approximately 7% of all term newborns and is even more common in cases of mild to moderate prematurity. In our study, 29 (52.7%) of 55 neonates with bradypnea (respiratory rate <30 breaths per minute) died, whereas only 1 (2%) of 49 neonates with tachypnea succumbed. Various pulmonary and extrapulmonary conditions can lead to tachypnea in neonates, but with progressive exhaustion, this can deteriorate into bradypnea at a much later stage. Delayed recognition of this transition, combined with prolonged transport duration, may contribute to the high mortality observed among neonates with poor respiratory effort or bradypnea.

Evidence-based research suggests that predictors with the highest impact on neonatal mortality, such as temperature instability, respiratory effort, and circulatory status, can be mitigated through optimal transport protocols. These include prereferral stabilization, intratransport monitoring, temperature maintenance, and effective communication between referring and receiving centers. These findings are congruent with our study. Furthermore, our analysis indicates that the SNS is a simple yet powerful tool with good discriminatory ability for mortality among sick neonates and may be especially beneficial for those transported from distant locations, even with transportation durations exceeding one hour.

The present study has several important limitations. Being a single-center observational study, the findings may not be generalizable to other settings. The SNS was not externally validated or calibrated, and inter-observer reliability was not assessed. Logistic regression analysis was exploratory and unadjusted for potential confounders such as illness severity or referral bias. Additionally, outcomes were limited to in-hospital discharge or death, without long-term follow-up. Henceforth, these factors restrict causal inference and warrant cautious interpretation of the findings.

## Conclusions

The present study highlights that the majority of neonates referred to our hospital were LBW infants, delivered via cesarean section and transported primarily through government ambulance services. The major indications for referral included respiratory distress, neonatal jaundice, sepsis, and perinatal asphyxia. These conditions necessitate effective temperature regulation, close monitoring, and appropriate management of respiratory distress and circulatory compromise both prior to and during neonatal transport. The SNS demonstrates strong association and good discriminative ability for in-hospital mortality among extramural neonates in a resource-limited setting.
